# The Inconveniences of Being a Good Fellow

**Published:** 1868-08-01

**Authors:** 


### *The Inconveniences of being a Good Fellow.*

The sweetest milk of human kindness will sometimes cream with righteous wrath, even though it may not acidulate in expression. All doctors are pre-supposed to be, *ex officio*, good fellows, and their offices appropriate places for other good fellows to congregate. Cigars, meerschaums, politics, gossip and tobacco-spittle pollute their ante-rooms, and even the inner *sanctum* is invaded. If a patient be not actually by the consulting chair, the *good fellow* is supposed to have nothing to do, and he is expected to devote his attention to helping empty-headed idlers to kill *their* time. The *good fellow* is to listen at all times, in genial mood, to tedious and long spun out narrations of imaginary ailments, and take his reward in additional notes to the continuous refrain which sounds his amiable disposition. As he is, of course, of charitable nature, he must attend all the poor in his neighborhood free of charge, whilst those able to pay are neglected, to be seized upon by Dr. Crusty over the way. Or, as usually happens, his own *able* friends will fail to call upon him in paying cases, because he is so kindly disposed, that he is anxious to have even his rival succeed. When Dr. Crusty has secured all the patient's money, and utterly failed to afford relief, the dilapidated carcass and empty pocket-book are straightway to be found in the ante-rooms of the *good fellow*. When Dr. Crusty has a paying case which he fails to relieve, he puts on a smoother face and asks *good fellow* his opinion and what to do. If benefit results, Dr. Crusty pockets the credit and the fee—if the issue is not favorable, the chance for a paid consultation or subsequent charge of the case, is foreclosed by the grave announcement: "I have already had the advice of the *good fellow*."

When a case is treated and the bill at last sent in, it is the last paid, " For, you know, the doctor is a *good fellow*, and won't mind it."

No one goes near *Crusty* for a subscription or any charitable purpose. *Good fellow's* office is always thronged like a town meeting for the benefit of the poor.

By a *good fellow*, herein, is not meant one who, in Yankee parlance, is styled " a *clever fellow*," an easy going, unsophisticated, little more than half witted chap; but a man with a head full of brains and information, which he gives away for the asking to those who either lack the former, or are too lazy to work for the latter. He can not say No! although his friends (?) are all the time robbing him of a capital more valuable than gold. Ten to one, should misfortune overtake him, these creatures will say: " He's a *good fellow*—BUT "—and a shrug and a wink are the sluice gates of a flood of calumny, or, at best, of depreciation.

Young Æsculapian friend—for Heaven's sake don't seek the reputation of being a *good fellow*.

